# Supplementary material for: A rat epigenetic clock recapitulates phenotypic aging and co-localizes with heterochromatin
Source: eLife. 2020 Nov 12;9:e59201. doi: 10.7554/eLife.59201 (PMC7661040; doi:10.7554/eLife.59201)
Supplement: Supplementary file 1. [file elife-59201-supp1.docx]

**Supplementary File 1**

**Table S1: PC1 Loading for Phenotypic Variables**

| Variable | PC1 Loading |
| --- | --- |
| open field - time ambulatory (s) | 0.3966 |
| open field - horizontal activity count | 0.3872 |
| open field - distance traveled (cm) | 0.3674 |
| open field - vertical activity count | 0.3646 |
| open field - time climbing (s) | 0.3629 |
| rotarod - mean time | 0.2325 |
| rotarod - max time | 0.2299 |
| open field - time in center (s) | 0.1729 |
| open field - time in stereotypy (s) | 0.0569 |
| open field - time resting (s) | -0.3915 |

**Table S2: Phenotypic Associations with DNAmAge based on Overlapped CpGs**

|  | | Beta Coefficient (P-value) | | |
| --- | --- | --- | --- | --- |
|  | | Model 1 | Model 2 | Model 3 |
| DNAmAge (PC1) | |  |  |  |
|  | Age | 0.54 (4.3e-15) | 0.45 (3.9e-12) | 0.29 (2.6e-4) |
|  | Pheno PC1 | -- | 0.43 (4.7e-4) | 0.39 (7.0e-4) |
|  | FACS PC1 |  | -- | 0.31 (1.5e-2) |
| Pheno PC1 and FACS PC1 are standardized variables, such that they have a standard deviation of 1. Given that the DNAmAge variable is in units of (months), the beta coefficients represent the increase in DNAmAge (in units of months) associated with every 1 s.d. increase in either PC1 FACS or PC1 Pheno. | | | | |

**Table S3: Association Between DNAmAge and Caloric Restriction (CR) in C57BL/6**

|  | | Beta Coefficient (P-value) | |
| --- | --- | --- | --- |
|  | | Model 1 | Model 2 |
| DNAmAge~ | |  |  |
|  | Age | 0.16 (<2e-16) | 0.16 (<2e-16) |
|  | CR | -1.21 (1.6e-4) | 1.18 (2.2e-1) |
|  | Age*CR | -- | -0.12 (9.1e-3) |

**Table S4: Phenotypic Associations with DNAmAge based on Overlapped CpGs**

|  | | Beta Coefficient (P-value) | | | |  |
| --- | --- | --- | --- | --- | --- | --- |
|  | | Green Module | Blue Module | Purple Module | Pink Module |  |
| DNAmAge (Module) | |  |  |  |  |  |
|  | Age | 0.32 (2.8e-2) | 0.04 (2.1e-1) | 0.43 (1.8e-2) | 0.03 (6.3e-3) |  |
|  | Pheno PC1 | **0.51 (4.9e-3)** | **0.34 (3.1e-3)** | 0.22 (3.9e-1) | 0.01 (3.0e-1) |  |
|  | FACS PC1 | 0.40 (0.1e-1) | -0.30 (9.1e-1) | 0.38 (2.1e-1) | -0.04 (1.2e-2) |  |
| Pheno PC1 and FACS PC1 are standardized variables, such that they have a standard deviation of 1. Given that the DNAmAge variable is in units of (months), the beta coefficients represent the increase in DNAmAge (in units of months) associated with every 1 s.d. increase in either PC1 FACS or PC1 Pheno. The grey module is not shown given that it is not a true module, but rather, consists of unassigned CpGs | | | | |  |  |

**Table S5: Association Between DNAmAge and Individual Phenotype Variables**

| Variable | Beta Coefficient | P-Value |
| --- | --- | --- |
| Distance Travelled | -1.70E-03 | **1.35E-03** |
| Horizontal Activity | -9.60E-04 | **4.80E-04** |
| Vertical Activity | -5.90E-03 | **5.30E-03** |
| Time Climbing | -1.30E-02 | **2.30E-02** |
| Time Resting | 5.00E-03 | **8.90E-03** |
| Time Ambulatory | -4.80E-03 | **9.90E-03** |
| Time in Center | -4.50E-03 | 4.30E-01 |
| Time in Stereotypy | 1.30E-03 | 9.40E-01 |
| Rotarod Mean | -2.50E-02 | 3.00E-01 |
| Rotarod Max | -5.00E-02 | **1.10E-02** |

*Results are from individual OLS models, adjusting for age and FACS PC1-5*
