# Supplementary material for: A rat epigenetic clock recapitulates phenotypic aging and co-localizes with heterochromatin
Source: eLife. 2020 Nov 12;9:e59201. doi: 10.7554/eLife.59201 (PMC7661040; doi:10.7554/eLife.59201)
Supplement: Transparent reporting form [file elife-59201-transrepform.docx]

***eLife’s* transparent reporting form**

We encourage authors to provide detailed information *within their submission* to facilitate the interpretation and replication of experiments. Authors can upload supporting documentation to indicate the use of appropriate reporting guidelines for health-related research (see [EQUATOR Network](http://www.equator-network.org/%20)), life science research (see the [BioSharing Information Resource](https://biosharing.org/)), or the [ARRIVE guidelines](http://www.plosbiology.org/article/info:doi/10.1371/journal.pbio.1000412) for reporting work involving animal research. Where applicable, authors should refer to any relevant reporting standards documents in this form.

If you have any questions, please consult our Journal Policies and/or contact us: [editorial@elifesciences.org](mailto:editorial@elifesciences.org).

**Sample-size estimation**

- You should state whether an appropriate sample size was computed when the study was being designed
- You should state the statistical method of sample size computation and any required assumptions
- If no explicit power analysis was used, you should describe how you decided what sample (replicate) size (number) to use

Please outline where this information can be found within the submission (e.g., sections or figure legends), or explain why this information doesn’t apply to your submission:

The sample size used for this analysis was consistent with sample sizes used for other epigenetic clock developments in rodents. Given that age was uniformly distributed, this provided us with sufficient power to identify an aging signature. The sample sizes used for training and test are available in the results section and methods section.

**Replicates**

- You should report how often each experiment was performed
- You should include a definition of biological versus technical replication
- The data obtained should be provided and sufficient information should be provided to indicate the number of independent biological and/or technical replicates
- If you encountered any outliers, you should describe how these were handled
- Criteria for exclusion/inclusion of data should be clearly stated
- High-throughput sequence data should be uploaded before submission, with a private link for reviewers provided (these are available from both GEO and ArrayExpress)

Please outline where this information can be found within the submission (e.g., sections or figure legends), or explain why this information doesn’t apply to your submission:

We included six rats (biological replicates) in each age group spanning 1 to 27 months. Our analytic sample included 134 rats. Excluded samples represent animals that died or were euthanized prior to blood draw, those that did not pass quality control for bisulfite sequencing, and/or those without complete FACS, rotarod, or open field data. This information is available in the results and methods sections.

**Statistical reporting**

- Statistical analysis methods should be described and justified
- Raw data should be presented in figures whenever informative to do so (typically when N per group is less than 10)
- For each experiment, you should identify the statistical tests used, exact values of N, definitions of center, methods of multiple test correction, and dispersion and precision measures (e.g., mean, median, SD, SEM, confidence intervals; and, for the major substantive results, a measure of effect size (e.g., Pearson's r, Cohen's d)
- Report exact p-values wherever possible alongside the summary statistics and 95% confidence intervals. These should be reported for all key questions and not only when the p-value is less than 0.05.

Please outline where this information can be found within the submission (e.g., sections or figure legends), or explain why this information doesn’t apply to your submission:

Raw values are shown in Figure 1 (A and C); Figure 2; Figure 3; Figure 4; and Figure 5D. Data used to generate these figures is also available via Source Data. Descriptions of statistical tests can be found in the results section when reporting findings and in the figure legends.

The following statistical methods were used in this study:

Biweight Midcorrelation

Kruskal-Wallis

Elastic Net Penalized Regression

Hierarchical Clustering

OLS Regression

WGCNA

PCA

Cistrome Enrichment (Giggle Analysis)

(For large datasets, or papers with a very large number of statistical tests, you may upload a single table file with tests, Ns, etc., with reference to sections in the manuscript.)

**Group allocation**

- Indicate how samples were allocated into experimental groups (in the case of clinical studies, please specify allocation to treatment method); if randomization was used, please also state if restricted randomization was applied
- Indicate if masking was used during group allocation, data collection and/or data analysis

Please outline where this information can be found within the submission (e.g., sections or figure legends), or explain why this information doesn’t apply to your submission:

Rat samples were split into training and test based on age to ensure even distribution, yet a slightly larger range and sample size in training to reduce overfitting. For instance, rats ages 2m, 6m, 10m, 14m, 18m, 22m, and 26m were selected for the test set (n=32), while all others were included in the training set (1m, 3m, 4m, 5m, 7m, 8m, 11m, 12m, 13m, 15m, 16m, 17m, 19m, 20m, 21m, 23m, 24m, 25m, 27m). This can be found in the results and methods sections.

**Additional data files (“source data”)**

- We encourage you to upload relevant additional data files, such as numerical data that are represented as a graph in a figure, or as a summary table
- Where provided, these should be in the most useful format, and they can be uploaded as “Source data” files linked to a main figure or table
- Include model definition files including the full list of parameters used
- Include code used for data analysis (e.g., R, MatLab)
- Avoid stating that data files are “available upon request”

Please indicate the figures or tables for which source data files have been provided:

We have provided the overlapped DNAmAge estimate and trait data for both mouse and rat. This data was used to generate Figures 2, 2S1, 3, and Tables S2 and S3.
